# Midfacial toddler excoriation syndrome (MiTES): case series, diagnostic criteria and evidence for a pathogenic mechanism

**DOI:** 10.1093/bjd/ljae151

**Published:** 2024-04-09

**Authors:** Nivedita Sarveswaran, Yunisa Pamela, Akhila A N Reddy, Akash P Mustari, Anchala Parthasarathi, Anthony J Mancini, Anuradha Bishnoi, Arun C Inamadar, Bayanne Olabi, Fiona Browne, Gargi N Deshmukh, Kenneth McWilliam, Keshavamurthy Vinay, Sahana Srinivas, Samantha Ibbs, Sivakumar Natarajan, Vadlamudi R Rao, Vijay Zawar, Vykuntaraju K Gowda, Samiha S Shaikh, Celia Moss, Christopher G Woods, Ichrak Drissi

**Affiliations:** Cambridge Institute for Medical Research, University of Cambridge, Cambridge, UK; Cambridge Institute for Medical Research, University of Cambridge, Cambridge, UK; Department of Biomedical Sciences, Faculty of Medicine, Universitas Padjadjaran, Bandung, Indonesia; Dr. Anchala Skin Institute and Research Center, Hyderabad, India; Department of Dermatology, Venereology and Leprology, Postgraduate Institute of Medical Education and Research, Chandigarh, India; Dr. Anchala Skin Institute and Research Center, Hyderabad, India; Division of Dermatology, Department of Pediatrics, Ann & Robert H. Lurie Children’s Hospital of Chicago, Northwestern University Feinberg School of Medicine, Chicago, IL, USA; Department of Dermatology, Venereology and Leprology, Postgraduate Institute of Medical Education and Research, Chandigarh, India; Department of Dermatology, Shri BM Patil Medical College & Hospital, BLDE University, Bijapur, India; Department of Dermatology, Edinburgh Royal Infirmary, UK; Department of Paediatric Dermatology, Children’s Health Ireland (CHI) at Crumlin, Crumlin, Ireland; Genome Foundation, Hyderabad, India; Paediatric Neurology, Neurosciences Department, Royal Hospital for Children and Young People, Edinburgh, UK; Department of Dermatology, Venereology and Leprology, Postgraduate Institute of Medical Education and Research, Chandigarh, India; Department of Pediatric Dermatology, Indira Gandhi Institute of Child Health, Bangalore, India; Department of Paediatric Dermatology, Birmingham Children’s Women’s and Children’s NHS Foundation Trust, Birmingham, UK; The Newcastle Upon Tyne Hospitals NHS Foundation Trust, UK; Genome Foundation, Hyderabad, India; Department of Dermatology, Dr. Vasantrao Pawar Medical College and Research Center, Nashik, India; Department of Pediatric Neurology, Indira Gandhi Institute of Child Health, Bangalore, Karnataka, India; Cambridge Institute for Medical Research, University of Cambridge, Cambridge, UK; Department of Paediatric Dermatology, Birmingham Children’s Women’s and Children’s NHS Foundation Trust, Birmingham, UK; College of Medical and Dental Sciences, University of Birmingham, Birmingham, UK; Cambridge Institute for Medical Research, University of Cambridge, Cambridge, UK; Cambridge Institute for Medical Research, University of Cambridge, Cambridge, UK

## Abstract

**Background:**

*PRDM12* polyalanine tract expansions cause two different disorders: midfacial toddler excoriation syndrome (MiTES; itch with normal pain sensation associated with 18 homozygous alanines (18A); and congenital insensitivity to pain (CIP) with normal itch associated with 19 homozygous alanines (19A). Knowledge of the phenotype, genotype and disease mechanism of MiTES is incomplete. Why 18A vs. 19A PRDM12 can cause almost opposite phenotypes is unknown; no other polyalanine or polyglutamine tract expansion disease causes two such disparate phenotypes.

**Objectives:**

To assess the genotype and phenotype of nine new, nine atypical and six previously reported patients diagnosed with MiTES.

**Methods:**

Using cell lines with homozygous PR domain zinc finger protein 12 (PRDM12) containing 12 alanines (12A; normal), 18A (MiTES) and 19A (CIP), we examined PRDM12 aggregation and subcellular localization by image-separation confocal microscopy and subcellular fractionation Western blotting.

**Results:**

MiTES presents in the first year of life; in all cases the condition regresses over the first decade, leaving scarring. The MiTES phenotype is highly distinctive. Features overlapping with PRDM12 CIP are rarely found. The genotype–phenotype study of the *PRDM12* polyalanine tract shows that having 7–15 alanines is normal; 16–18 alanines is associated with MiTES; 19 alanines leads to CIP; and no clinically atypical cases of MiTES had a polyalanine tract expansion. PRDM12 aggregation and subcellular localization differed significantly between 18A and normal 12A cell lines and between 18A and 19A cell lines. MiTES is a new protein-aggregation disease.

**Conclusions:**

We provide diagnostic criteria for MiTES and improved longitudinal data. MiTES and CIP are distinct phenotypes, despite their genotypes varying by a single alanine in the PRDM12 polyalanine tract. We found clear distinctions between the cellular phenotypes of normal, MiTES and CIP cells. We hypothesize that the developmental environment of the trigeminal ganglion is unique and critically sensitive to pre- and postnatal levels of PRDM12.

## Teaser

This study investigated polyalanine tract expansions in *PRDM12*, with a focus on midfacial toddler excoriation syndrome (MiTES) and congenital insensitivity to pain (CIP). MiTES, presenting in the first year of life, exhibits a unique phenotype that regresses over a decade, leaving scarring. Analysis of polyalanine tract expansions reveals specific associations: 7–15 alanines is normal, 16–18 alanines is linked to MiTES with PRDM12 aggregation and 19 alanines leads to CIP with aggregated and mislocalized PRDM12 in the cytoplasm. In conclusion, MiTES is identified as a novel protein-aggregation disease and, despite a single alanine variation, MiTES and CIP are distinctly characterized phenotypes.

Linked Article: Has *Br J Dermatol* 2024; **191**:323–324.

Author Video: https://youtu.be/3tyliVQHAJ4

**What is already known about this topic?**

- Mid-facial toddler excoriation syndrome (MiTES) is a genetic cause of extreme dysaesthesia anatomically limited to the face.
- It presents in the first year of life and causes significant self-inflicted facial scarring.
- No treatments are known.
- Almost all cases of MiTES have a homozygous GCC repeat expansion in *PRDM12*, resulting in the N-terminal polyalanine tract expanding from the normal range of 7–13 alanines to 18 alanines.

**What does this study add?**

- In most cases, MiTES subsides after 5 years and scarring fades by the mid-20s.
- Apart from the face, pain and itch perception are normal.
- MiTES is a protein-aggregation disease caused by an expanded polyalanine repeat.
- The subcellular patterns of protein aggregation clearly differ between 18-alanine MiTES and 8–19-alanine hereditary autonomic and sensory neuropathy.
- *PRDM12* is the only human repeat expansion disease-causing gene known to cause two clearly distinct phenotypes.

**Figure 1:**
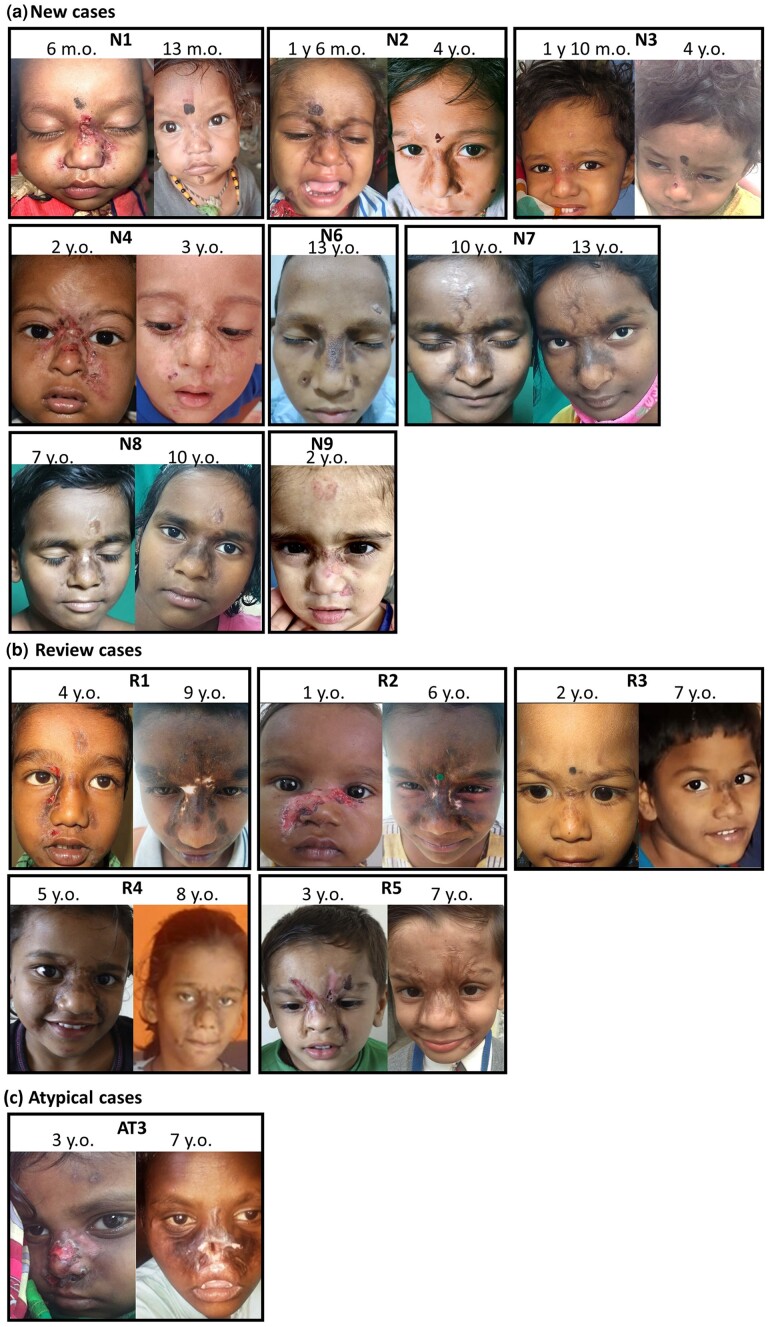
Facial features of mid-facial toddler excoriation syndrome (MiTES). (a) New patients with MiTES: N1 at 6 months and 13 months; N2 at 1 year 6 months and 4 years; N3 at 1 year 10 months and 4 years; N4 at 2 years and 3 years; N6 at 13 years; N7 at 10 years and 13 years; N8 at 7 years and 10 years; N9 at 2 years old. (b) Natural history of MiTES by review of cases: R1 at 4 years and 9 years; R2 at 1 year and 6 years; R3 at 2 years and 7 years; R4 at 5 years and 8 years; and R5 at 3 years and 7 years old. (c) Atypical cases where the facial features did meet MiTES diagnostic criteria but the *PRDM12* genotype was normal: AT3 at 3 years and 7 years old. See Table S2 for clinical details.

**Figure 2:**
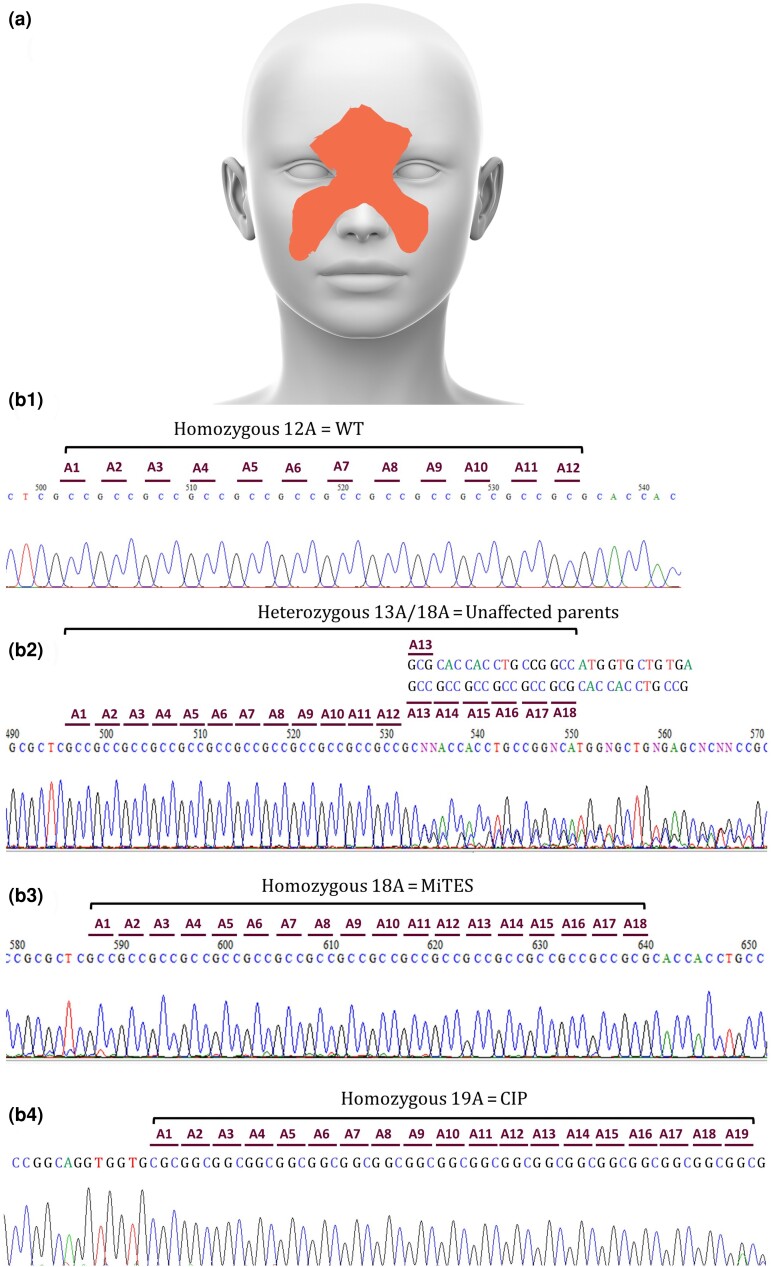
Facial areas affected by mid-facial toddler excoriation syndrome (MiTES) and electrophoretograms of an unaffected MiTES-carrier parent, a patient with MiTES and a patient with hereditary autonomic and sensory neuropathy 8 (HSAN8) congenital insensitivity to pain (CIP). (a) The affected facial regions in patients with MiTES are highlighted in orange. (b) Chromatogram traces serve as examples to illustrate the GC-rich nature of the polyalanine tract in exon 5 of *PRDM12*. (b1) Chromatogram trace of homozygous wild-type 12-alanine (12A) *PRDM12*. (b2) Chromatogram trace of heterozygous 13-alanine/18-alanine (13A/18A) *PRDM12* from a carrier parent of an affected child. (b3) Chromatogram trace of homozygous *PRDM12* 18A from a patient with MiTES. (b4) Chromatogram trace of homozygous *PRDM12* 19A from a patient with HSAN8/CIP. Reading the genetic sequence is relatively easy when the polyalanine tract length is homozygous. However, in cases where a frameshift occurs due to differences in the polyalanine tract between two alleles, such as those from an affected parent, the precise assignment of nucleotides at each site is essential.

Mid-facial toddler excoriation syndrome (MiTES) was first reported in 2017 as a novel phenotype following three cases presented at a UK dermatology meeting.^1^ Four subsequent reports comprising 12 further cases allowed the early phenotype to be defined as the onset of persistent facial self-excoriation in the first year of life leading to deep wounds and subsequent scarring but restricted to a characteristic mid-facial distribution (Figures 1, 2a).^1–5^ The authors noted the severity and unusualness of mutilating self-inflicted skin ulcers in children so young and in such a highly reproducible distribution across only the face that could not be explained by any known disease. Lesions to the depth of muscle were observed and extended in an X-like pattern across the bridge of the nose to the eyebrows and upper cheeks, while notably sparing the cornea (see Figure 2a). MiTES appeared to have an autosomal recessive inheritance pattern, with females and males being equally affected. In 2018, this was confirmed by the finding of homozygous expansions of the *PRDM12* polyalanine tract to 18 alanines.^2^

In 2015, *PRDM12* mutations were linked to congenital insensitivity to pain (CIP), resembling hereditary autonomic and sensory neuropathy types 4 and 5 (HSAN4 and HSAN5) and designated PRDM12-CIP or HSAN8 (Table S1; see Supporting Information).^6,7^ In all three conditions, the normal embryonic development of nociceptive sensory neurons fails.^7,8^ Most PRDM12-CIP variants are stop, splicing or null missense mutations. However, in the original paper, two families with several affected members had expanded *PRDM12* polyalanine tracts; family A showed a CIP phenotype with a homozygous 19-alanine expansion (19A); family J had features of MiTES in early childhood and, later, partial CIP with a homozygous 18-alanine expansion (18A).^1,7,9–11^

PR domain zinc finger protein 12 (PRDM12) is a transcription factor that controls the initiation and maintenance of tropomyosin receptor kinase A (TrkA) expression during the critical period of neural crest cell differentiation. Biallelic mutations in *PRDM12* disrupt the histone methylation of key genetic targets during embryogenesis, resulting in the complete absence of TrkA-positive sensory neurons from birth.^7,12,13^

This paper more completely defines the MiTES phenotype and genotype and its natural history; examines patients with atypical *PRDM12* findings; and reviews family J with MiTES and later-onset features of CIP. We show that MiTES is a disease caused by polyalanine-driven protein aggregation. Finally, we report a possible mechanism that results in two distinct phenotypes caused by near-identical polyalanine expansions in *PRDM12*: homozygous 18A in MiTES and homozygous 19A in CIP.^7,13^

## Patients and methods

### Phenotype

New cases with the characteristic self-inflicted mid-facial skin damage comprising deep excoriations, atrophic scars and/or postinflammatory hyperpigmentation were referred by dermatologists to C.M. and C.G.W. Other diagnoses had been eliminated and treatments had little effect. All clinical data, family history and any associated features were reviewed before and after *PRDM12* mutation screening.

We requested a phenotype review from the physicians on all previous cases. This included progression or regression of itch behaviours and scarring, of abnormal pain or itch sensation, general health and other illnesses, and symptoms of peripheral neuropathy (including skin ulcers, peripheral paraesthesia and anomalies of touch, temperature and pain sensation). We also requested recent facial images and – where patients were old enough – we asked for first-hand narratives about their symptoms.

### Genotype: *PRDM12* Sanger sequencing and molecular genetic studies

In our experience, both exomes and genomes are unreliable for detecting pathogenic *PRDM12* polyalanine tract expansions. Therefore, we used Sanger sequencing in the patients reported here.

Analysis of the *PRDM12* polyalanine tract was performed on the new families, including two patients who had previously undergone exome sequencing in commercial laboratories with normal *PRDM12* results. We compared the results with those for previous patients and controls.^2^

### Cell and molecular biologic studies

The full methodology is provided in Appendix S1 (see Supporting Information).

## Results

### Typical mid-facial toddler excoriation syndrome phenotype

We report nine new affected children (N1–N9) from eight families; all had features typical of MiTES [Figure 1, Table S2 (see Supporting Information)]. We reviewed six previously reported cases (R1–R6); all showed substantial healing but with significant scarring and often postinflammatory pigmentary changes (Figure 1). Combining new and reviewed cases allowed for more complete reporting of the phenotype (Figures 1, 2a). Common behaviours included persistent scratching, nose-pulling, slapping the face and disturbed sleep. Parents reported that this behaviour gradually abated during the first decade of life and scarring healed by the third decade, with patient-reported normal facial pruriception from the second decade onward. Affected individuals remembered the itch as powerful, continuous, overwhelming and impossible to ignore; that scratching caused pain, which relieved the itch; and that the itch gradually subsided during the first decade and had resolved by the second decade.^2^ The abnormal sensation was not reported elsewhere on the body. There was no evidence of reduced pain sensation elsewhere on the body, with no reports of lip, tongue, inner cheek or corneal injuries, or deficiencies in sweating or lacrimation, as would be expected in any form of CIP, including PRDM12-CIP.^6^ Particularly noteworthy is the lack of severe and deep facial (and elsewhere) *Staphylococcus aureus* infections, despite the constant itching and skin injuries; repeated significant *S. aureus* infections are an obligatory feature of Mendelian disorders of congenital nociceptor deficiency, including PRDM12-CIP/HSAN8.^6^

### Atypical phenotype: mid-facial toddler excoriation syndrome with features of congenital insensitivity to pain

Apart from the facial lesions, manifestations of altered pain sensation have not usually been reported in MiTES. An exception is the five siblings previously reported as family J,^7^ of whom the youngest four had diabetes-like foot ulcers and the eldest three had abnormal sensory nerve conduction results; the youngest two did not. In all five, MiTES has subsided, leaving scars. None complain of sensory problems, such as tingling of the extremities, and none consider themselves to have abnormal sensation. However, their mother maintains that they do have peripheral insensitivity to pain: one had hand blisters from carrying hot items and all can easily develop traumatic ulcers on their feet (cases ATJ1–5 in Table S2).

### Atypical phenotype: mid-facial toddler excoriation syndrome associated with Frey syndrome

One child was initially diagnosed as having bilateral congenital Frey syndrome but developed typical features of MiTES during his first year (ATF in Table S2) with homozygous *PRDM12* 18A. Now aged 7 years, his facial itch has reduced and facial scars are slowly improving. Frey syndrome with blushing and occasionally sweating on the cheeks (in the typical Frey distribution and not in the typical MiTES facial distribution) continues, particularly provoked by hot and spicy foods. His parents and sibling do not have Frey syndrome or features of MiTES. No other patient with MiTES that we have tested has had Frey syndrome.

A sibling pair with congenital bilateral Frey syndrome has been previously reported. We confirmed that they had no MiTES features; no facial itch or scarring of the face, and they report no other problems.^14^ Their parents were unaffected, unrelated and White.

### Atypical mid-facial toddler excoriation syndrome with no *PRDM12* mutations

We reviewed the three patients (AT1–AT3) referred to us as possibly having MiTES but who had clinically atypical features and normal *PRDM12* gene analysis (including the polyalanine tract) [see Figure 1 (AT3 not shown) and Table S2]. All three cases were sporadic. In two (AT1 and AT2), the onset of MiTES was after the age of 1 year and the areas affected were not as severe as in MiTES.

AT3 had extremely severe and persistent MiTES with facial mutilation (Figure 1), associated with unexplained severe global developmental delay, principally cognitive. Growth and general health were normal, with no other neurological findings. There was no relevant family history.

### Genotype: *PRDM12* Sanger sequencing

All new cases were homozygous for the *PRDM12* 18A repeat (Figure 2b). Almost all previously reported individuals with MiTES were also *PRDM12* 18A homozygotes (Table S2); one sibling pair was suspected of being hemizygous for the 18A repeat,^2^ and one child was a compound heterozygote for 17A/18A repeats.^2^ A further child was reported by Noguera-Morel *et al*. as heterozygous for 16A/18A repeats with a high pain threshold but no other features of CIP.^4^

The parents, most of whom were tested, were all heterozygotes for a normal and a disease-causing allele (16A, 17A, 18A and a suspected gene deletion) – none had any feature of MiTES.^4,7^ One carrier mother was heterozygous for 15A/18A repeats and did not have MiTES during childhood, confirming that 15 alanines is a benign *PRDM12* polyalanine expansion.

In the previously reported family with congenital bilateral Frey syndrome,^14^ one (asymptomatic) parent and both affected girls had a heterozygous seven-base pair deletion in the terminal exon of *PRDM12* (c.717delCGCGGGC p.Pro239Profs228*). This deletion has not been previously reported in gnomAD (https://gnomad.broadinstitute.org) or dbSNP (https://www.ncbi.nlm.nih.gov/projects/SNP/get_html.cgi?whichHtml=overview). Genome sequencing revealed no further *PRDM12* variants in any family member. By using single nucleotide polymorphisms within the 0.5 Mb of chromosome 9 centred on *PRDM12* we found that the siblings had inherited different maternal alleles (data not shown).

### Eighteen- and 19-alanine mutations induce aggregation of PRDM12 in HEK293 cells

**Figure 3:**
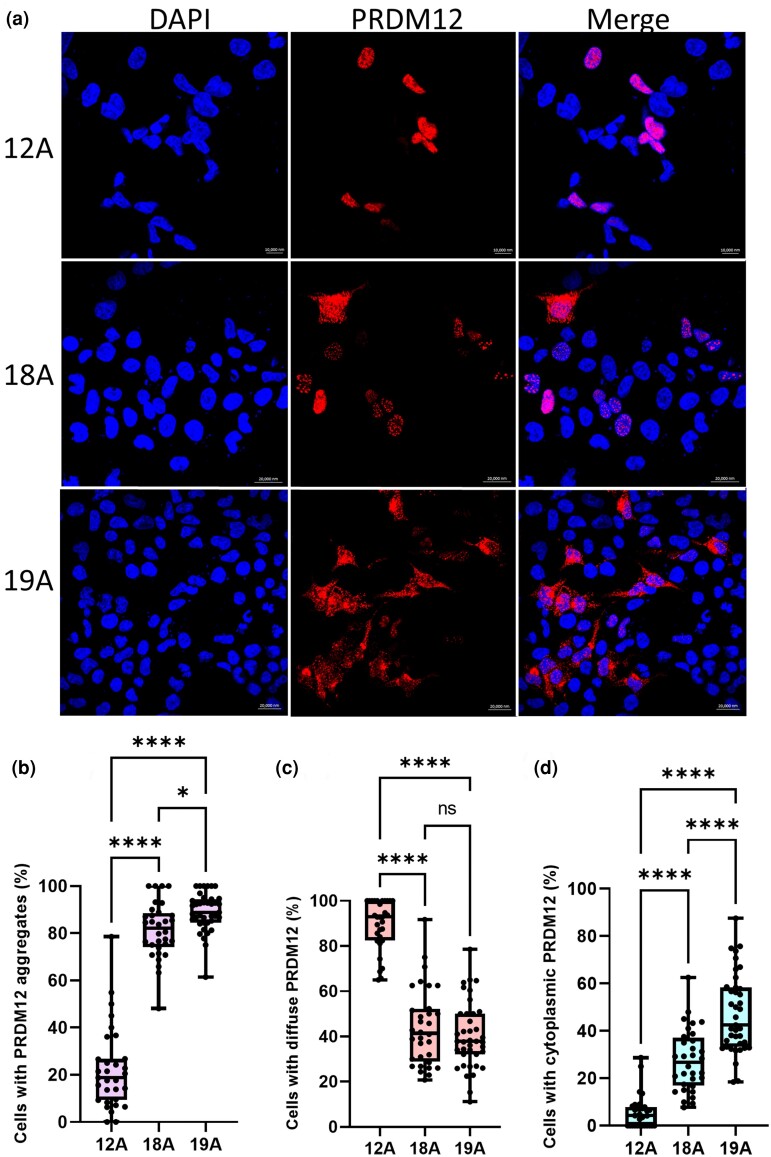
Cellular phenotypes and subcellular localizations of wild-type (WT) 12-alanine (12A) PR domain zinc finger protein 12 (PRDM12) and mutant 18- (18A) and 19-alanine (19A) proteins. (a) WT PRDM12 12A was expressed diffusely in the nucleus (red channel, upper panel) and co-localized with 4ʹ,6-diamidino-2-phenylindole (DAPI; blue channel). The PRDM12 18A form representing the mid-face toddler excoriation syndrome (MiTES) condition produced small but bright nuclear aggregates in addition to diffuse nuclear staining (red channel, second row). Cytoplasmic aggregates were also observed. The PRDM12 19A form representing HSAN8/CIP formed larger plaques in the nucleus and the cytoplasm with less diffuse expression of PRDM12 in the nucleoplasm, as though more protein had been drawn up into aggregates (red channel, third row). (b) The percentage of cells exhibiting aggregated PRDM12 was calculated for each image. Cells transfected with *PRDM12* 18A or *PRDM12* 19A displayed a notably higher percentage of cells exhibiting aggregated PRDM12 than those transfected with *PRDM12* 12A. In particular, PRDM12 19A exhibited a higher degree of aggregation than PRDM12 18A. (c) The percentage of cells exhibiting diffuse PRDM12 was lower in cells transfected with mutant *PRDM12* 18A or 19A than those transfected with the WT. (d) The percentage of cells expressing both PRDM12 18A and 19A forms in the cytoplasm was higher than that of WT PRDM12 12A, with PRDM12 19A showing a significantly higher percentage of expressing cells than PRDM12 18A. All graphs display minimum to maximum whiskers and each datapoint represents the percentage of cells from one image from three independent experiments (12A, *n* = 31; 18A, *n* = 32; 19A, *n* = 39). The line in the box indicates the median. ns, nonsignificant, one-way Anova with Tukey’s post-hoc test. *P < 0.05 ; ****P < 0.0001.

Human influenza haemagglutinin (HA)-tagged *PRDM12* fusion constructs were transiently transfected into HEK293 cells to investigate differences in subcellular localization between the wild-type (WT; 12A), MiTES (18A) and CIP (19A) forms of PRDM12. Cells transfected with *PRDM12* WT/12A produced homogeneous PRDM12 staining in the nucleus, with low-level diffuse staining in the cytoplasm (Figure 3a). Expression of the 18A and 19A constructs produced a spectrum of cellular phenotypes, including prominent bright nuclear PRDM12 aggregates of variable size and cytoplasmic mislocalization (Figure 3a). Neither of these phenotypes were seen in WT-transfected cells. In contrast to WT PRDM12, the fluorescence intensity of 18A and 19A nuclear aggregates was stronger and did not specifically localize to the nucleus, as indicated by 4ʹ,6-diamidino-2-phenylindole staining (Figure 3a). This pattern was broadly similar between mutant constructs; however, where nuclear spots of similar size were expressed, 19A-transfected cells appeared to have less diffuse expression of PRDM12 in the nucleoplasm, as though more protein had been drawn up into these aggregates (Figure 3a). Mutant PRDM12 localized in the cytoplasm formed fibril-like patterns extending from the nuclear envelope to the cell perimeter with small clumps. In more dramatic cases, mutant PRDM12 formed larger plaques in the nucleus and cytoplasm. Immunofluorescence analysis was used to determine the percentage of cells exhibiting either diffuse or abnormally aggregated PRDM12, with the count performed per image. Cells transfected with 18A or 19A *PRDM12* constructs exhibited a higher mean (SD) percentage of cells displaying PRDM12 nuclear aggregates than those transfected with WT [12A: 22.3% (17.4), *n* = 31; 18A: 81.2% (11.8), *n* = 32; 19A: 89.3% (8.4), *n* = 39 (one-way Anova F(2,99) = 270.5; *P* < 0.001)]. Specifically, cells transfected with 19A constructs showed a higher percentage of aggregation than those transfected with 18A (*P* < 0.05; Figure 3b). The mean (SD) percentage of cells that exhibited diffuse PRDM12 was higher with 12A constructs than with 18A and 19A ones [12A: 89.4% (11.1), *n* = 31; 18A: 43.9% (17.1), *n* = 32; 19A: 40.3% (14.7), *n* = 39 (one-way Anova F(2,99) = 114.9; *P* < 0.001)]. Notably, no statistically significant difference was found between cells expressing the 18A and 19A constructs (*P* > 0.05; Figure 3c).

### Eighteen- and 19-alanine mutations induce cytoplasmic mislocalization of PRDM12 in HEK293 cells

The same immunofluorescence approach was employed to assess the subcellular localization of PRDM12. Regardless of the form of PRDM12, the protein consistently localized to the nucleus (data not shown) with PRDM12 12A rarely found in the cytoplasm. However, the mean (SD) percentage of cells exhibiting cytoplasmic PRDM12 was highest for 19A vs. 18A and 12A [12A: 5.6% (7.0), *n* = 31; 18A: 27.4% (13.0), *n* = 32; 19A: 47.2% (16.6), *n* = 39 (one-way Anova F(2,99) = 85.7; *P* < 0.001)] (Figure 3d). We then used HEK293 cell fractionation to further assess the immunofluorescence results (see Appendix S1). Western blot analysis and protein quantification indicated that PRDM12 12A exhibited the highest expression in the whole-cell lysate (Figure S1a, b; see Supporting Information), but no significant difference was found between any of the groups. After the fractionation process, PRDM12 19A exhibited a higher relative expression in the cytoplasm than 18A and 12A. Also, PRDM12 19A displayed the lowest relative expression in the nuclear fraction when compared with 18A and 12A, resulting in the highest cytoplasmic/nuclear ratio for PRDM12 19A (see Figure S1b2, b3, b4).

## Discussion

MiTES has a highly consistent phenotype, as confirmed by the new and previously reported cases reviewed here. MiTES is characterized by severe self-inflicted, highly localized symmetrical facial excoriation commencing between 3 and 6 months of age and slowly diminishing over the first decade.^1^ MiTES results in severe facial scarring, which improves after the first decade, albeit often with noticeable pigmentary changes and atrophy (Figure 1). It seems likely that the abnormal scratching behaviour is a response to extreme itch or dysaesthesia, possibly with co-localized insensitivity to pain, as has been suggested in trigeminal trophic syndrome. The absence of self-inflicted injuries elsewhere implies a normal pain sensation in the unaffected areas of face and the rest of the body. We propose diagnostic criteria for MiTES (Table S2) and a flowchart outlining the procedural approach to patients presenting with MiTES (Figure S2; see Supporting Information).

**Figure 4:**
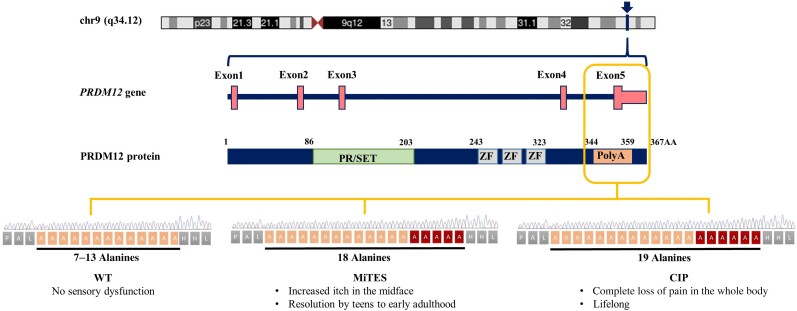
Graphic illustration of human *PRDM12*, PRDM12 protein, polyalanine tract (Poly A) and the human phenotype of biallelic polyalanine expansion mutations. The figure shows the *PRDM12* locus on chromosome 9, the gene and the protein architecture. Amino acid numbering is shown along the top of the PRDM12 protein schematic representation. Depending on the number of alanines in the polyalanine tract, the phenotype might be different (7–13 alanines: no sensory dysfunction; 18 alanines: mid-facial toddler excoriation syndrome; 19 alanines: congenital insensitivity to pain). PR/SET, PR domain; WT, wild type; ZF, zinc finger motif.

MiTES can be clinically distinguished from other neurological or infectious causes of chronic itch or repetitive self-mutilating behaviour (e.g. trigeminal trophic syndrome, erythropoietic protoporphyria and Lesch–Nyhan syndrome), from known Mendelian forms of itch (reported with particular mutations in *SCN9A*, *SCN11A* and *COL6A1*),^15–17^ and can be confirmed by genetic testing.^1,2,5^ The most frequent genetic finding in MiTES is a homozygous expansion of the *PRDM12* polyalanine tract to 18A repeats. Expansions to 16A/18A and 17A/18A give an identical MiTES phenotype, and we hypothesize that 16A/17A would as well, but no cases have yet been described (Figure 4).^4,18,19^

The presence of peripheral ulcers in affected older members of a previously reported family homozygous for *PRDM12* 18A prompted us to ask if progressive nociceptor neuropathy could be part of the MiTES phenotype.^7^ However, none of the other affected individuals of a comparable age has any features of a neuropathy. We have no explanation for this discrepancy; ongoing follow-up will be necessary to see if a nociceptive neuropathy develops.

Of the three patients referred to us with a possible diagnosis of MiTES but without *PRDM12* mutations, AT1 and AT2 had minimal excoriations with no scarring and therefore we concluded on clinical grounds that they did not have MiTES. In the third patient (AT3), the MiTES features were typical but more severe than in any other case, with structural damage to the nose, and associated with significant learning difficulties, suggesting that MiTES may be genetically heterogeneous.

We have studied one child with congenital bilateral Frey syndrome and MiTES who had the typical homozygous *PRDM12* 18A mutation, and one of the authors has previously seen another patient with both diagnoses (but no molecular genetic analysis). However, we specifically sought Frey syndrome features in all new and reviewed patients, and these were absent. Given the rarity of both conditions, this co-occurrence is likely to be a real but rare association. Here, we have also reported the molecular genetics of siblings with congenital Frey syndrome who we confirmed did not have any features of MiTES. A very rare *PRDM12* deletion was found, but extensive family studies allowed us to conclude that it was benign. Investigations of the three patients with congenital Frey syndrome together did not reveal a shared genetic aetiology.

We found that *PRDM12* constructs representing the MiTES (18A) and CIP (19A) expansion proteins both formed nuclear aggregates when expressed in HEK293 cells, which were rare with the WT (12A) protein. This is consistent with *in vitro* findings from other transcription factors whose polyalanine expansions are associated with disease.^20,21^ Given these results, PRDM12 MiTES should also be considered a polyalanine expansion disorder, likely to be causing disease through abnormal protein aggregation. Additionally, PRDM12 18A and 19A expansion proteins were found to be expressed in the cytoplasm, which, again, was rare with WT 12A.

Aggregation and cytoplasmic mislocalization are common *in vitro* features of polyalanine expansion disorders, although pathogenicity *in vivo* is attributed to mutant protein degradation.^22^*PRDM12* expansions associated with CIP are likely to present a similar loss-of-function mechanism at the cellular level, where the absence of nucleoplasmic PRDM12 precludes the development of nociceptors in trigeminal and dorsal root ganglia. In contrast, the partial nuclear availability of mutant PRDM12 in MiTES may have a dosage effect on transcriptional cascades that depend on precise local concentrations at specific embryonic time points, thus altering the intrinsic identity of select somatosensory neurons. Sensory dysfunction could arise from misexpression of signal-transducing receptors, molecules involved in neurotransmission or defects in peripheral and/or central innervation.

*PRDM12* is expressed only in peripheral nociceptors and pruriceptors, so these specialized neurons seem likely to account for the contrasting *PRDM12* phenotypes: 18A (MiTES) and 19A (HSAN8).^12–24^ Regarding nociceptors, our studies suggest that, in HSAN8, the 19A mutation sequesters PRDM12, essentially making it nonfunctional and resulting in absent pain sensation, while in MiTES, the 18A mutation allows enough PRDM12 for nociceptor genesis in most of the head and body.^12,23–25^ Itch sensation is preserved in HSAN8, suggesting that the requirement for PRDM12 in pruriceptor development is less than for nociceptors so the 18A mutation allows for normal itch sensation in most of the head and body. However, this does not explain the mid-facial localization in MiTES of both abnormal itch sensation and possibly reduced pain sensation.

In MiTES, the distribution of lesions across the mid-face is highly reproducible and coincides with the central regions of overlap of the ophthalmic (V1) and maxillary (V2) branches of the trigeminal sensory nerve. The trigeminal ganglion, a unique sensory ganglion, contains both motor and sensory neurons and receives two separate sets of sensory neuron precursors that contribute to the development of nociceptors, originating from both the neural crest (similar to dorsal root ganglia for peripheral nociceptors and pruriceptors) and trigeminal cranial placodes.^26^ Whether PRDM12 regulates different transcription programmes in the trigeminal cranial placodes population, as has been shown for its downstream signal Brn3a, is unknown.^27^ The MiTES phenotype strongly suggests that 18A mutation results in hyperexcitable pruriceptors, but only within the trigeminal ganglia. This could imply that the 18A, but not the 19A mutation, may be partially functional through an unknown mechanism.^28–33^ The precise reason behind the spectrum of the MiTES phenotype remains unclear. It is possible that environmental factors such as hot weather and the consumption of spicy food might exacerbate the phenotype. A further conundrum is why MiTES resolves by the teenage years. Some older patients feel that they have learned to control their response to itch, but it does seem that the itch sensation subsides; the neurological or cutaneous mechanism for this remains unknown.

Currently, there is no specific treatment for MiTES. Topical antistaphylococcal antimicrobials are advisable. There may be a role for *N*-acetylcysteine given its apparent effectiveness in treating trigeminal trophic syndrome.^34^ Our research indicates the potential for topical *PRDM12* gene therapy to alleviate early itching and prevent later facial scarring in MiTES.

## Supplementary Material

ljae151_Supplementary_Data

## Data Availability

The data underlying this article are available in the article and in its online Supporting Information.
