# Supplementary material for: Midfacial toddler excoriation syndrome (MiTES): case series, diagnostic criteria and evidence for a pathogenic mechanism
Source: Br J Dermatol. 2024 Apr 9;191(3):437–46. doi: 10.1093/bjd/ljae151 (PMC11324070; doi:10.1093/bjd/ljae151)
Supplement: ljae151_Supplementary_Data [file ljae151_supplementary_data.zip › Appendix S1.docx]

**Appendix S1 Supplement Methodology**

**Genotype: *PRDM12* Sanger Sequencing**

Genomic DNA was isolated from suspected cases, parents and affected relatives where possible. Sequencing primers were designed using the reference transcript NM_021619.2. Exons 1 – 4 were amplified using the KOD Hot Start DNA Polymerase kit (Sigma-Aldrich), while exon 5 was amplified using FailSafe PCR 2X PreMix J (Lucigen) with OneTaq DNA Polymerase (NEB). PCR products were purified by enzymatic treatment with Exonuclease I and Shrimp Alkaline Phosphatase (both from NEB). In all cases, patient sequences were compared to the reference transcript NM_021619.2 using the alignment software, BioEdit version 7.2.6.1. Where expansion variants were found in affected cases, parents were sequenced to determine whether they were inherited or a result of *de* *novo* mutation.

***PRDM12* Plasmid Preparation**

Wild-type and mutant *PRDM12* plasmids were synthesised by Source BioScience. The coding sequence was based on the reference transcript NM_021619.2, with an N-terminal fusion human influenza hemagglutinin (HA) tag, placed within the pcDNA3 mammalian expression vector. The polyalanine tract length was modified according to clinical phenotype and most common genotype: wild-type 12 alanine repeat (12A), MiTES (18A) and CIP (19A).

**HEK293 Cell Maintenance**

HEK293 cells were maintained in high-glucose DMEM with pyruvate and GlutaMAX Supplement (Gibco), supplemented with 10% fetal bovine serum (Sigma-Aldrich). Cells were grown in 75-cm^2^ tissue culture treated flasks, incubated at 37°C and 5% CO_2_, and split at a 1:10 ratio twice a week.

**Nucleus/Cytoplasm Fractionation**

HA-tagged PRDM12 fusion constructs were transiently transfected into HEK293 cells to elucidate the subcellular localization patterns of PRDM12 within the cellular context. The subsequent subcellular fractionation process was conducted to isolate distinct cellular compartments, namely the cytoplasmic, membrane and organelle, and nuclear and cytoskeletal (Nuc) fractions, in accordance with the manufacturer's guidelines provided in the Cell Fractionation Kit (Cell Signaling Technology, USA). All preparations were performed on ice. Transfected cells were harvested washed with cold PBS and resuspended in 0.5 ml of cold PBS. Cells were counted using trypan blue staining and automated cell counter. 100ul aliquot of cell suspension was retained for whole cell lysate (WCL). The remaining 400µl were centrifuged at 500xg at 4˚C for 5 minute and the appropriate volume of CIB buffer was added and centrifuged at 5 minutes at 500xg. The resulting supernatant was saved as the cytoplasmic fraction. The remaining pellet was resuspendend in the appropriate volume of membrane isolation buffer (MIB), then centrifuged for 5 minutes at 8,000xg. The supernatant from this centrifugation was saved as the membrane fraction. For the final steps, the pellets were resuspended in the appropriate volume CyNIB buffer followed by three times of sonication for 5 seconds each to produce the nuclear fraction. Five µl of protease inhibitor cocktail provided by the kit was added to every 500µl of each subcellular fraction.

**Immunofluorescence microscopy and data analysis**

HEK293 cells were cultured on poly-L-lysine coated coverslips and transfected with PRDM12 (12A) or (18A) or (19A) constructs using FuGENE HD (Promega) according to the manufacturer’s protocol at a ratio of 3:1. 24 hours after transfection, cells were fixed by 10 min incubation in 4% paraformaldehyde. Cells were permeabilized by 10 minute incubation in 0.1% PBS-Triton X-100. Permeabilisation buffer was removed before directly adding normal goat serum 10% for blocking (Invitrogen) for 30 minutes at room temperature. Cells were stained using PRDM12 primary antibody (Atlas). Secondary antibody used was Alexa Fluor 546 goat anti-rabbit (Invitrogen). Images were acquired with an LSM880 confocal microscope (Zeiss). ZEN software (Zeiss) was used for the data analysis. From three independent experiments, thirty or more images with five or more transiently transfected cells were randomly used. The number of cells with diffuse or abnormally aggregated PRDM12 as well as the number of cells with cytoplasmic or nuclear PRDM12 were determined for each image. The counted number of cells was normalised to the total number of transfected cells for each image and percentages of cells with diffuse, aggregated, cytoplasmic and nuclear PRDM12 were calculated. To minimise the bias, PRDM12 aggregation and localisation were analysed by two individuals, while a third person conducted a blind analysis. The average of data analysis was used for statistical analysis.

**Western Blot Analysis**

The whole cell lysate was obtained by lysing cells in RIPA buffer. Ten µg of protein were taken from whole cell lysate, cytoplasmic fraction or nuclear fraction and separated using NuPAGE^TM^ 4 to 12% Bis-Tris (Invitrogen). Primary antibodies are anti-PRDM12 (ATLAS) (4:1000) and anti-karyopherin alpha (BD Bioscience) (1:1000). Secondary antibodies are purchased from Cell Signalling and signal detected using the enhanced chemiluminescence (ECL, Amersham) western blot analysis system. Quantification of Western blot bands was conducted using ImageJ. All PRDM12 bands chemiluminescence intensities were first normalized to the corresponding karyopherin alpha band. Subsequently, PRDM12 bands from both the cytoplasmic and nuclear fractions were further normalized to the corresponding band in the whole cell lysate.
