# Supplementary material for: Midfacial toddler excoriation syndrome (MiTES): case series, diagnostic criteria and evidence for a pathogenic mechanism
Source: Br J Dermatol. 2024 Apr 9;191(3):437–46. doi: 10.1093/bjd/ljae151 (PMC11324070; doi:10.1093/bjd/ljae151)
Supplement: ljae151_Supplementary_Data [file ljae151_supplementary_data.zip › Tables S1, S2.docx]

**Table S1:** Genetic mutations associated with congenital insensitivity to pain.

HSAN, Hereditary sensory and autonomic neuropathies; NTRK1, Neurotrophic receptor tyrosine kinase 1; NGF, nerve growth factor

|  | Gene | Known mutations | Reference |
| --- | --- | --- | --- |
| HSAN4 | NTRK1 | p.G517E  p.G522E  p.L657P  p.I699T  p.C752S  p.C763S  p.R771C | *Shaikh et al.,2017* |
| HSAN5 | NGF | c.661C→T  c.[680C>A]+[681_682delGG]  p.R121W | *Einarsdottir et al., 2004*  *Carvalho et al.,2011*  *Shaikh et al.,2018* |
| HSAN8 | PRDM12 | Asp31Tyr  Ser58Lysfs*85  Ile102Asn  Trp160Cys  Arg168Cys  Glu172Asp  c.683-1G>A  His289Leu  Ala354_Ala359dup  Ala353_Ala359dup | *Chen et al.,2020* |

**References**

Shaikh SS, Chen Y, Halsall S, Nahorski MS, Omoto K, Young GT, Phelan A, Woods CG. A comprehensive funstional analysis of NTRK1 missense mutations causing hereditary sensory and autonomic neuropathy type IV(HSAN IV). Hum Mutat. 2017; 38(1): 55-63

[Einarsdottir](https://pubmed.ncbi.nlm.nih.gov/?term=Einarsdottir+E&cauthor_id=14976160) E, [Carlsson](https://pubmed.ncbi.nlm.nih.gov/?term=Carlsson+A&cauthor_id=14976160) A, [Minde](https://pubmed.ncbi.nlm.nih.gov/?term=Minde+J&cauthor_id=14976160) J, [Toolanen](https://pubmed.ncbi.nlm.nih.gov/?term=Toolanen+G&cauthor_id=14976160) G, [Svensson](https://pubmed.ncbi.nlm.nih.gov/?term=Svensson+O&cauthor_id=14976160) O, [Solders](https://pubmed.ncbi.nlm.nih.gov/?term=Solders+G&cauthor_id=14976160) G, [Holmgren](https://pubmed.ncbi.nlm.nih.gov/?term=Holmgren+G&cauthor_id=14976160) G, [Holmberg](https://pubmed.ncbi.nlm.nih.gov/?term=Holmberg+D&cauthor_id=14976160) D, [Holmberg](https://pubmed.ncbi.nlm.nih.gov/?term=Holmberg+M&cauthor_id=14976160) M. A mutation in the nerve growth factor beta gene (NGFB) causes loss of pain perception. Hum Mol Genet. 2004 Apr 15;13(8):799-805.

[Carvalho](https://pubmed.ncbi.nlm.nih.gov/?term=Carvalho+OP&cauthor_id=20978020) OP,  [Thornton](https://pubmed.ncbi.nlm.nih.gov/?term=Thornton+GK&cauthor_id=20978020) GK, [Hertecant](https://pubmed.ncbi.nlm.nih.gov/?term=Hertecant+J&cauthor_id=20978020) J, [Houlden](https://pubmed.ncbi.nlm.nih.gov/?term=Houlden+H&cauthor_id=20978020) H, [Nicholas](https://pubmed.ncbi.nlm.nih.gov/?term=Nicholas+AK&cauthor_id=20978020) AK, [Cox](https://pubmed.ncbi.nlm.nih.gov/?term=Cox+JJ&cauthor_id=20978020) JJ, [Rielly](https://pubmed.ncbi.nlm.nih.gov/?term=Rielly+M&cauthor_id=20978020) M, [Al-Gazali](https://pubmed.ncbi.nlm.nih.gov/?term=Al-Gazali+L&cauthor_id=20978020) L, [Woods](https://pubmed.ncbi.nlm.nih.gov/?term=Woods+CG&cauthor_id=20978020) CG. A novel NGF mutation clarifies the molecular mechanism and extends the phenotypic spectrum of the HSAN5 neuropathy. J Med Genet. . 2011 Feb;48(2):131-5.

[Shaikh](https://pubmed.ncbi.nlm.nih.gov/?term=Shaikh+SS&cauthor_id=30296891) SS, [Nahorski](https://pubmed.ncbi.nlm.nih.gov/?term=Nahorski+MS&cauthor_id=30296891) MS, [Woods](https://pubmed.ncbi.nlm.nih.gov/?term=Woods+CG&cauthor_id=30296891) CG. A third HSAN5 mutation disrupts the nerve growth factor furin cleavage site. Mol Pain. 2018; 14: 1744806918809223.

**Supplementary Table 2:** Phenotype and genotype data of the new (N1 to N9), reviewed (R1 to R6), and atypical cases (AT1 to AT3, ATF and ATJ1 to ATJ5) referred to the authors as Midfacial Toddler Excoriation syndrome (MiTES)

| **ID** | **Country of origin** | **Sex** | **Age at firs presentation** | **Age when first seen** | **Age when reviewed** | **Main features** | **Persistent scarring** | **Associated features** | **Family history** | ***PRDM12* genotype** | **Previous publication** |
| --- | --- | --- | --- | --- | --- | --- | --- | --- | --- | --- | --- |
| **New cases** | | | | | | | | | | | |
| N1 | Karnataka, India | F | 10 m.o. | 13 m.o. | NA | Excoriations over bridge of nose gradually progressed to involve to the perinasal region | Yes | Excessive irritability and sleep disturbances | - Consanguineous parents, uncle, niece - No family history of MiTES - 2 well older sisters | 18A/18A | NA |
| N2 | Karnataka, India | F | 9 m.o. | 1 y 6 m.o. | 4 y.o. | Excoriations | Yes | Epilepsy | NA | 18A/18A | NA |
| N3 | Karnataka, India | M | 4 m.o | 1 y 10 m.o. | 4 y.o. | Excoriations | Yes | - Global developmental delay - Previously rubbing of hands and feet - Lichenification present on dorsum of hands and feet | NA | 18A/18A | NA |
| N4 | India | M | 4 m.o. | 2 y.o. | 3 y.o. | Facial scratching | Yes | None | - Parents were 3rd cousins - Well elder sibling | 18A/18A | NA |
| N5 | Karnataka, India | M | 9 m.o | 10 y.o. | 6 m.o. | Mid-face excoriations | Yes | Episode of encephalitis aged 19 months | - Consanguineous parents - Normal elder sibling | 18A/18A | NA |
| N6 | Karnataka, India | M | 4 y.o. | 13 y.o. | NA | Severe excoriation | Yes | None | - NA | 18A/18A | NA |
| N7 | Maharashtra, India | F | 5 m.o. | 10 y.o. | 13 y.o. | Itch with excoriations on nose, central face and forehead, improving on review | Yes | - Right hand and left leg tingling   On follow up: pain seemed normal | - Consanguineous parents - N7 is sibling of N8 | 18A/18A | NA |
| N8 | Maharashtra , India | F | 5 m.o. | 7 y.o. | 10 y.o | Excoriations on nose, central face and forehead, improving on review | Yes | - Normal temperature sensing - Right hand and left leg tingling - On follow up: pain seemed normal | - Consanguineous parents - N8 is sibling of N7 | 18A/18A | NA |
| N9 | Punjab, India | F | 6 m.o. | 2 y.o. | NA | Repeated scratching around the eyes, nose and forehead | Yes | None | - No parental consanguinity - Well elder sister | 18A/18A | NA |
| **Reviewed case** | | | | | | | | | | | |
| R1 | Karnataka, India | M | 9 m.o. | 4 y.o. | 9 y.o. | At first presentation:   - Facial excoriations   On review:   - Scratching on and off on mid face - Symptom free for longer periods (months) | Yes | None | - Consanguineous parents - Older and younger sisters affected - R1 is sibling of R2 | 18A/ Exon 5 deletion | Moss *et* *al*, 2018^2^ |
| R2 | Karnataka, India | F | 4 m.o. | 1 y.o. | 6 y.o. | At first presentation:   - Facial excoriations   On review:   - Scratching on and off on mid face - Symptom free for longer periods (months) | Yes | None | - Consanguineous parents - Older sister and brother also affected - R2 is sibling of R1 | 18A/ Exon 5 deletion | Moss *et* *al*, 2018^2^ |
| R3 | Karnataka, India | M | 1 y.o. | 2 y.o. | 7 y.o. | At first presentation:   - Facial excoriations   On review:   - Scratching has stopped | Yes | None | - Consanguineous parents - A cousin was reported to have same skin lesions during childhood | 18A/18A | Moss *et* *al*, 2018^2^ |
| R4 | Karnataka, India | F | 6 m.o. | 5 y.o. | 8 y.o. | At first presentation:   - Midfacial skin lesions   On review:   - Scratching has stopped | Yes | On review:   - Itching on elbows and knees – no erosions | - No parental consanguinity - R4 is sibling of R5 | 18A/ 18A | Inamadar *et al*, 2019^3^ |
| R5 | Haryana, India | M | 6 m.o. | 3 y.o. | 7 y.o. | At first presentation:   - Midfacial skin lesions   On review:   - Scratching has stopped | Yes | On review:   - Itching on elbows and knees – no erosions | - No parental consanguinity - R5 is sibling of R4 | 18A/ 18A | Inamadar *et al*, 2019^3^ |
| R6 | Haryana, India | M | 1 y.o. | 3 y.o. | 12 y.o. | At first presentation:   - Midfacial excoriations and nose-pulling   On review:   - Scratching has stopped | Yes | At first presentation:   - Delayed development   On review:   - Normal development, attending school | - NA | Not done | Srinivas *et al*, 2017^1^ |
| **Atypical case** | | | | | | | | | | | |
| AT1 | British Caucasian | F | 1 y.o. | 18 m.o. | 4 y.o. | At first presentation:   - Few isolated excoriations on cheeks   On review:   - Single small persisting lesion | No | None | - NA | 12A/12A | NA |
| AT2 | British Caucasian | F | 8 m.o. | 2 y.o. | NA | Facial rubbing with discrete, punched-out lesions across forehead and nasal bridge | No | None | - NA | 13A/13A and no other mutation was found in *PRDM12* | NA |
| AT3 | Karnataka, Indian | M | 1 y.o. | 3 y.o. | 7 y.o. | At first presentation:   - Midfacial excoriations with rubbing and slapping of the affected area   On review:   - Progression of skin lesions with mutilation of the nose, hyperpigmentation and atrophy of the mid face | Yes | Severe global delay, on treatment with Risperidone | - Third degree related parents | 12A/12A | Moss *et* *al*, 2018^2^ |
| ATF | British Caucasian | M | 3 m.o | 4 y.o. | 8 y.o. | At first presentation;  - Bilateral cheek erythema on breast feeding, and then during weaning  On review:   - Scratching reduced | Yes | On review:  Frey’s feature remain as before | - NA | 18A/18A | NA |
| ATJ1 | Irish Caucasian | M | 3 m.o. | 19 y.o. | 28 y.o. | At first presentation:   - Facial excoriations   On review:  Scratching has stopped | Yes | On review:  No skin ulcers on the feet | - ATJ1 – ATJ5 are siblings | 18A/18A | Chen *et al*. 2015^7^ |
| ATJ2 | Irish Caucasian | M | 3 m.o. | 15 y.o. | 24 y.o. | At first presentation:   - Facial excoriations   On review:   - No facial itch | Yes | On review:  Skin ulcers on the feet | - ATJ1 – ATJ5 are siblings | 18A/18A | Chen *et al*, 2015^7^ |
| ATJ3 | Irish Caucasian | M | 3 m.o. | 13 y.o. | 22 y.o. | At first presentation:   - Facial excoriations   On review:   - Scratching has stopped | Yes | On review:  Skin ulcers on the feet | - ATJ1 – ATJ5 are siblings | 18A/18A | Chen *et al*, 2015^7^ |
| ATJ4 | Irish Caucasian | F | 3 m.o. | 11 y.o. | 19 y.o. | At first presentation:   - Facial excoriations   On review:   - Scratching has stopped | Yes | On review:  Skin ulcers on the feet | - ATJ1 – ATJ5 are siblings | 18A/18A | Chen *et al*, 2015^7^ |
| ATJ5 | Irish Caucasian | F | 3 m.o. | 11 y.o. | 19 y.o. | At first presentation:   - Facial excoriations   On review:   - Scratching has stopped | Yes | On review:  Skin ulcers on the feet | - ATJ1 – ATJ5 are siblings | 18A/18A | Chen *et al*, 2015^7^ |
