# Supplementary figures and images for: Midfacial toddler excoriation syndrome (MiTES): case series, diagnostic criteria and evidence for a pathogenic mechanism
Source: Br J Dermatol. 2024 Apr 9;191(3):437–46. doi: 10.1093/bjd/ljae151 (PMC11324070; doi:10.1093/bjd/ljae151)

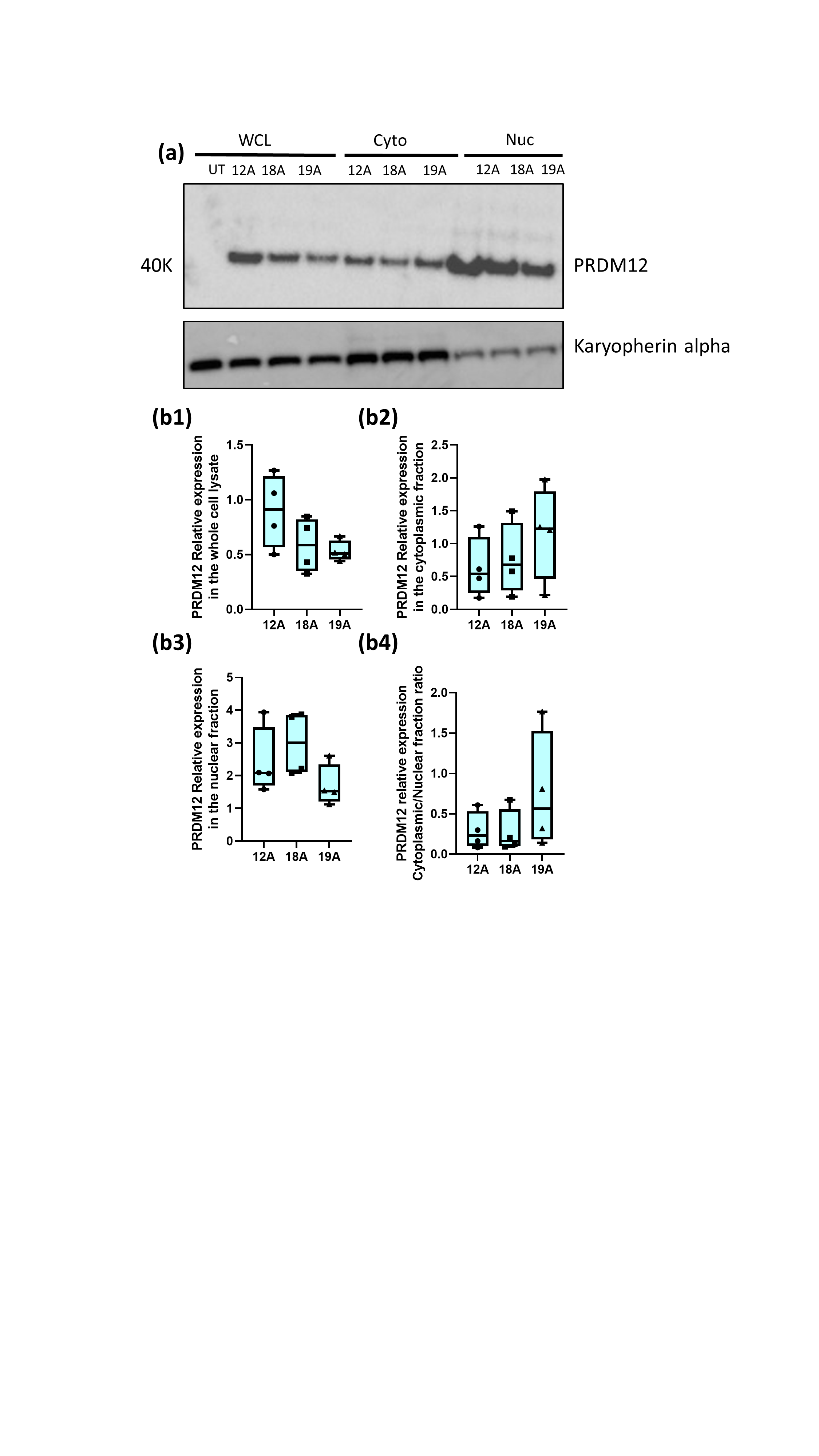

Supplement: ljae151_Supplementary_Data [file ljae151_supplementary_data.zip › Figure S1_edited.PNG]

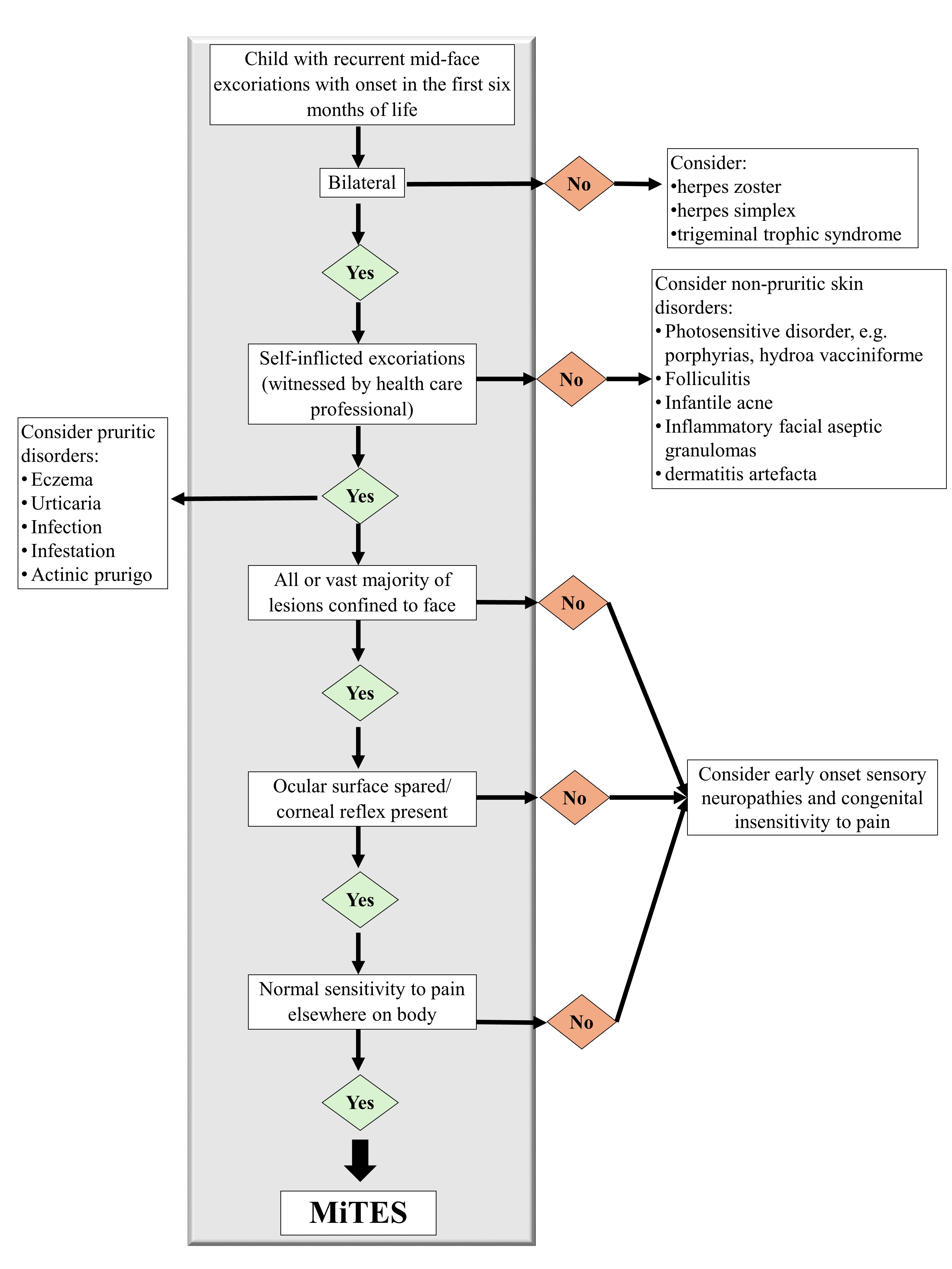

Supplement: ljae151_Supplementary_Data [file ljae151_supplementary_data.zip › Figure S2.JPG]
